# Crystal structure of deglycosylated human IgG4-Fc

**DOI:** 10.1016/j.molimm.2014.05.015

**Published:** 2014-11

**Authors:** Anna M. Davies, Roy Jefferis, Brian J. Sutton

**Affiliations:** a King's College London, Randall Division of Cell and Molecular Biophysics, New Hunt's House, London SE1 1UL, United Kingdom; b Medical Research Council & Asthma UK Centre in Allergic Mechanisms of Asthma, London, United Kingdom; c University of Birmingham, College of Medical & Dental Sciences, School of Immunity & Infection, Edgbaston, Birmingham B15 2TT, United Kingdom

**Keywords:** Degly-Fc, deglycosylated human IgG4-Fc, G(0), agalactosyl glycoform, (G2F)_2_, glycoform comprising Gal β1→4 GlcNAc β1→2 Man α1→6 (Gal β1→4 GlcNAc β1→2 Man α1→3) Man β1→4 GlcNAc β1→4 (α1–6 Fuc) GlcNAc, (MN2F)_2_, glycoform comprising Man β1→4 GlcNAc β1→4 (α1→6 Fuc)GlcNAc, Antibody, Immunoglobulin, Glycosylation, Fc receptor, Fc–Fc interaction, Complement

## Abstract

The Fc region of IgG antibodies, important for effector functions such as antibody-dependent cell-mediated cytotoxicity, antibody-dependent cellular phagocytosis and complement activation, contains an oligosaccharide moiety covalently attached to each C_H_2 domain. The oligosaccharide not only orients the C_H_2 domains but plays an important role in influencing IgG effector function, and engineering the IgG-Fc oligosaccharide moiety is an important aspect in the design of therapeutic monoclonal IgG antibodies. Recently we reported the crystal structure of glycosylated IgG4-Fc, revealing structural features that could explain the anti-inflammatory biological properties of IgG4 compared with IgG1. We now report the crystal structure of enzymatically deglycosylated IgG4-Fc, derived from human serum, at 2.7 Å resolution. Intermolecular C_H_2-C_H_2 domain interactions partially bury the C_H_2 domain surface that would otherwise be exposed by the absence of oligosaccharide, and two Fc molecules are interlocked in a symmetric, open conformation. The conformation of the C_H_2 domain DE loop, to which oligosaccharide is attached, is altered in the absence of carbohydrate. Furthermore, the C_H_2 domain FG loop, important for Fcγ receptor and C1q binding, adopts two different conformations. One loop conformation is unique to IgG4 and would disrupt binding, consistent with IgG4's anti-inflammatory properties. The second is similar to the conserved conformation found in IgG1, suggesting that in contrast to IgG1, the IgG4 C_H_2 FG loop is dynamic. Finally, crystal packing reveals a hexameric arrangement of IgG4-Fc molecules, providing further clues about the interaction between C1q and IgG.

## Highlights

• The first crystal structure of deglycosylated human IgG4-Fc is reported at 2.7 Å resolution.
• The asymmetric unit comprises a novel interlocked arrangement of two IgG4-Fc molecules.
• The C_H_2 domains are oriented in an “open” arrangement.
• The structure of the C_H_2 domain DE loop is altered in the absence of carbohydrate.
• Crystal packing reveals a hexameric Fc arrangement.

## Graphical abstract

**Figure:**
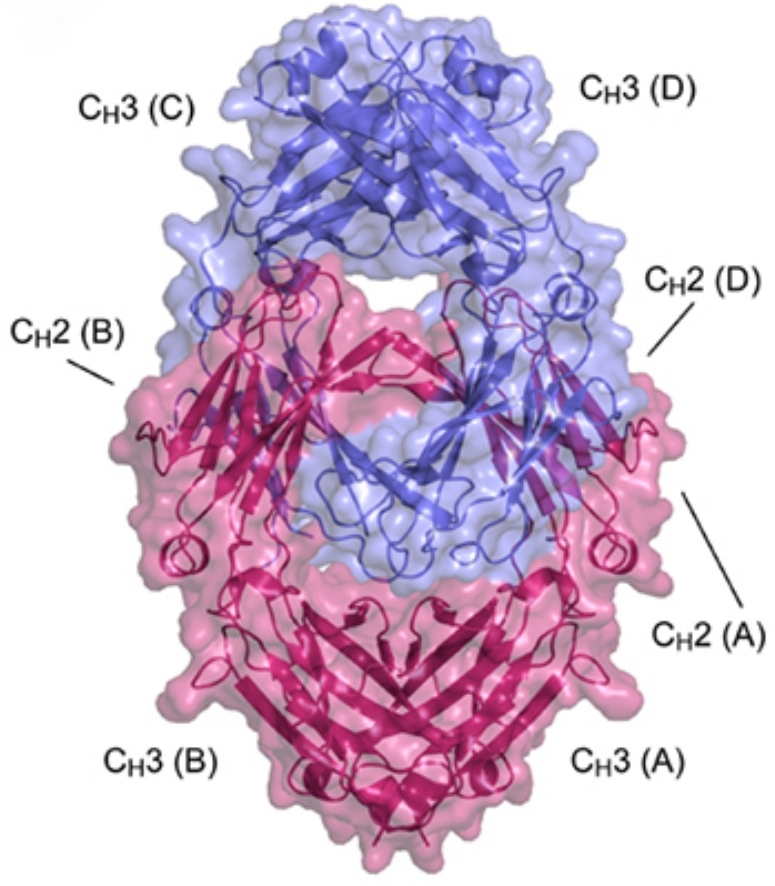

## 1 Introduction

IgG antibodies are responsible for antibody-dependent cell-mediated cytotoxicity, antibody-dependent cellular phagocytosis and complement activation, functions mediated through binding of the Fc region to Fcγ receptors and C1q, respectively. The Fc region comprises a dimer of C_H_2 and C_H_3 domains that are linked to the two Fab fragments through the hinge region, and an oligosaccharide moiety covalently attached to the C_H_2 domain at position 297. The typical pattern of glycosylation is that of a heptasaccharide bi-antennary core, which can additionally contain fucose, galactose and sialic acid residues (Jefferis, 2009, Jefferis, 2012). Altered IgG glycosylation plays a role in disease (Jefferis, 2012). For example agalactosylation is found in Crohn's disease and rheumatoid arthritis, where the level of galactosylation is negatively correlated with disease severity (Tomana et al., 1988, Parekh et al., 1989).

The oligosaccharide not only orients the C_H_2 domains (Krapp et al., 2003) but affects Fcγ receptor binding and complement activation (Kanda et al., 2006, Ferrara et al., 2011, Forthal et al., 2010), and therefore plays an important role in influencing IgG effector function. Engineering the IgG-Fc oligosaccharide moiety is thus an attractive approach in the design of therapeutic monoclonal IgG antibodies (Abès and Teillaud, 2010, Jefferis, 2012).

Crystal structures have been solved for human IgG1-Fc containing the typical bi-antennary moiety (e.g. Idusogie et al., 2000, Matsumiya et al., 2011), in addition to glycoforms modified through enzymatic activity or manipulation of the carbohydrate processing pathway (Krapp et al., 2003, Crispin et al., 2009, Bowden et al., 2012). Crystal structures have also been solved for human IgG1-Fc and murine IgG1-Fc devoid of oligosaccharide (Feige et al., 2009, Borrok et al., 2012; Braden, DOI:10.2210/pdb3dnk/pdb).

Recently, we reported the crystal structures of serum-derived and recombinant human IgG4-Fc, revealing structural features that could explain the unique, anti-inflammatory biological properties of IgG (Davies et al., 2014). We now report the crystal structure of enzymatically deglycosylated, serum-derived human IgG4-Fc (degly-Fc)*, solved at 2.7 Å resolution.

The deglycosylated IgG4-Fc structure reveals two interlocked Fc molecules in a novel packing arrangement, with the C_H_2 domains oriented in a symmetric, open conformation. The part of the C_H_2 domain surface exposed by the absence of oligosaccharide is partially buried by intermolecular C_H_2-C_H_2 domain interactions. The degly-Fc structure also reveals conformational differences in functionally important loop regions. The structure of the C_H_2 DE loop, containing residue 297, is altered in the absence of oligosaccharide. The C_H_2 FG loop, which is involved in Fcγ receptor and C1q binding, adopts two different conformations. One conformation is unique to IgG4 and would disrupt Fcγ and C1q binding, while the second is similar to the conserved conformation found in IgG1, folded back on to the C_H_2 domain, suggesting that the IgG4 C_H_2 FG loop is dynamic. Recently, involvement of an IgG hexamer in the interaction with C1 was reported (Diebolder et al., 2014). Crystal packing in the degly-Fc structure reveals a hexameric arrangement of IgG4-Fc molecules that involves movement of the C_H_2 domain AB loop, providing further insight into the structural requirements for the IgG/C1q interaction.

## 2 Materials and methods

### 2.1 Protein preparation and crystallisation

Serum-derived, human IgG4-Fc (degly-Fc)*, derived from the Rea IgG4 myeloma protein (Jefferis et al., 1990), was enzymatically deglycosylated as described previously (Ghirlando et al., 1999). Crystals were grown at 291 K using a reservoir of 50 μL 100 mM Bis-Tris propane pH 7.5, 20% (w/v) PEG 3350 and 200 mM sodium citrate, and a drop size of 100 nL protein (6 mg/mL) and 200 nL reservoir. Crystals were harvested after 2 months and briefly cryoprotected in a solution of 100 mM Tris–HCl pH 7.0, 20% (w/v) PEG 3350, 200 mM sodium citrate, and 20% (v/v) glycerol before flash-cooling in liquid nitrogen.

### 2.2 Structure determination, model building and refinement

Data were collected at beamline I04-1 at the Diamond Light Source (Harwell, UK). Integration was performed with XDS within the *xia2* package (Kabsch, 2010, Winter, 2010), and further processing was carried out using the CCP4 suite (Winn et al., 2011). The structure was solved by molecular replacement with MOLREP (Vagin and Teplyakov, 1997) using protein atoms from PDB accession number 4C54 (Davies et al., 2014). Refinement was performed with PHENIX (Adams et al., 2010) and manual model building with *Coot* (Emsley et al., 2010). TLS groups were assigned with PHENIX. Overall structure quality was assessed with MolProbity (Chen et al., 2010) and POLYGON (Urzhumtseva et al., 2009) within PHENIX. Buried surface areas were calculated with CNS (Brünger et al., 1998). Data processing and refinement statistics are presented in Table 1. Coordinates and structure factors have been deposited in the Protein Data Bank with accession number 4D2N. Figures were produced with PyMOL (The PyMOL Molecular Graphics System, Version 1.1r1, Schrödinger, LLC). C_H_2 domain loops (AB, BC, DE and FG) are referred to in accordance with the C1-type immunoglobulin domain strand definition of Halaby et al. (1999).

**Table 1:** Data processing and refinement statistics.

Data processing	
Space group	*P*622
Unit cell dimensions	
*a*, *b*, *c* (Å)	196.95, 196.95, 96.96
Resolution (Å)	53.68–2.70 (2.83–2.70)^a^
No. of unique reflections^b^	30 902 (4 012)^a^
Completeness (%)^b^	99.9 (99.9)^a^
Redundancy^b^	20.0 (20.0)^a^
Mean ((*I*)/σ(*I*)) b	16.9 (1.6)^a^
*R*_pim_ (%)^b^	4.0 (61.2) a
Wilson *B* factor (Å^2^)	64.7

Refinement	
*R*_work_/*R*_free_ (%)	20.45/25.44
RMSD	
Bond lengths (Å)	0.002
Bond angles (°)	0.603
Coordinate error (Å)	0.38
No. of atoms	
Protein	6310
Solvent	12
Other^d^	6
Ave. *B* factor (Å^2^)	
Protein: C_H_2 A/B/C/D	81.8/84.5/92.6/87.8
Protein: C_H_3 A/B/C/D	56.8/56.9/73.8/79.8
Solvent	54.9
Other^d^	91.0
Ramachandran plot^c^	
Favoured (%)	98.3
Allowed (%)	100

a Numbers in parentheses are for the highest resolution shell.

b Data scaled with Aimless (Winn et al., 2011, Evans and Murshudov, 2013).

c Ramachandran plot generated by MolProbity (Chen et al., 2010).

d Glycerol.

## 3 Results and discussion

### 3.1 Overall structure and molecular packing

**Fig. 1:**
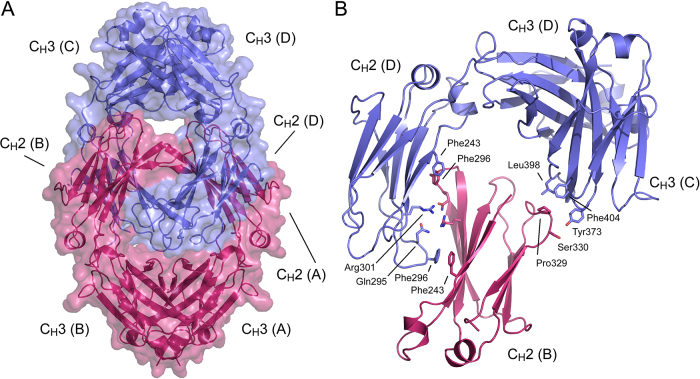
Overall structure. (A) The two interlocked Fc molecules of the asymmetric unit (blue and pink) are shown, centred on the intermolecular C_H_2-C_H_2 interaction between chains B and D. The overall packing is such that intermolecular C_H_2-C_H_2 and C_H_2-C_H_3 interactions for chain A are with chains C and D, chain B with chains D and C, chain C with chains A and B, and chain D with chains B and A, respectively. (B) Detailed view of the interaction between chains B, C and D. C_H_2-C_H_2 contacts are formed between chains B and D by residues Phe243, Gln295, Phe296 and Arg301. C_H_2-C_H_3 contacts are formed between chains B and C, respectively, by residues Pro329, Ser330, Tyr373, Leu398 and Phe404. (For interpretation of the references to colour in this figure legend, the reader is referred to the web version of this article.)

The asymmetric unit of the deglycosylated IgG4-Fc (degly-Fc)* structure contains two interlocked Fc molecules related to one another by a pseudo-symmetric two-fold rotation (Fig. 1A). No interpretable electron density was present for residues preceding Gly236, Pro238, Gly237 or Leu235 for chains A, B, C and D, respectively. Superposition of IgG structures containing at least one intact hinge disulfide bond (e.g. Mizushima et al., 2011) on either molecule of the degly-Fc structure revealed atomic clashes between the hinge and the second interlocked molecule. Given the orientation of the two interlocked molecules, and that SDS-PAGE analysis of the degly-Fc protein revealed the hinge region was not intact in all Fc molecules in the sample (data not shown), it is possible that the species lacking an intact hinge was selectively crystallised.

The overall orientation of C_H_2 and C_H_3 domains is essentially identical for all four chains, which could be superposed with r.m.s. deviations of 0.39–0.90 Å. While there are local differences at the interfaces between the four chains of the degly-Fc asymmetric unit, some due to side chain disorder, the general features can be described as follows. The C_H_2 domain from chain A simultaneously contacts the C_H_2 domain from chain C and the C_H_3 domain from chain D. The overall molecular packing is such that C_H_2-C_H_2 and C_H_2-C_H_3 domain interactions for chain B are with chains D and C, those for chain C are with chains A and B, and those for chain D are with chains B and A, respectively, with an average buried surface area of 1470 Å^2^.

Because of some side chain disorder in chain A, a detailed description of the intermolecular C_H_2-C_H_2 and C_H_2-C_H_3 interfaces is presented from the perspective of chain B (Fig. 1B): The C_H_2-C_H_2 domain interaction between chains B and D has pseudo two-fold symmetry, and comprises residues forming hydrogen bonds (Gln295 and Arg301), flanked by others forming van der Waals interactions (Phe243 and Phe296). The C_H_2-C_H_3 domain interface between chains B and C is formed predominantly from van der Waals interactions. This interface comprises C_H_2 domain FG loop residues Pro329 and Ser330 (chain B), and Lys340, Tyr373, Leu398 and Phe404 (chain C) (Fig. 1B).

With the exception of conversion of Asn297 to Asp297 through the activity of PNGase F, and conformational differences in loop regions (described below), some due to the absence of oligosaccharide, there were no significant differences between the overall structure of deglycosylated IgG4-Fc and glycosylated IgG4-Fc (Davies et al., 2014).

### 3.2 C_H_2 domain surface

IgG typically contains a heptasaccharide bi-antennary core, with additional fucose, galactose and sialic acid residues (Jefferis, 2009). The serum-derived Rea myeloma protein used for this study has a 70% G(0)* (agalactosyl) oligosaccharide moiety (Jefferis et al., 1990), but was enzymatically deglycosylated (Ghirlando et al., 1999), and thus no electron density was observed for any carbohydrate.

In glycosylated IgG4-Fc, the heptasaccharide core covers the surface of the C_H_2 domain, burying a total area of ∼1000 Å^2^. The patch exposed by the absence of carbohydrate in the degly-Fc structure is partially covered by the intermolecular C_H_2-C_H_2 domain interface, burying a total surface area of ∼750 Å^2^. While the surface buried by the C_H_2-C_H_2 domain interface is not identical to that buried by carbohydrate, residues Phe243 and Arg301 participate in both C_H_2-C_H_2 domain and C_H_2–carbohydrate interactions.

### 3.3 C_H_2 domain orientation

**Fig. 2:**
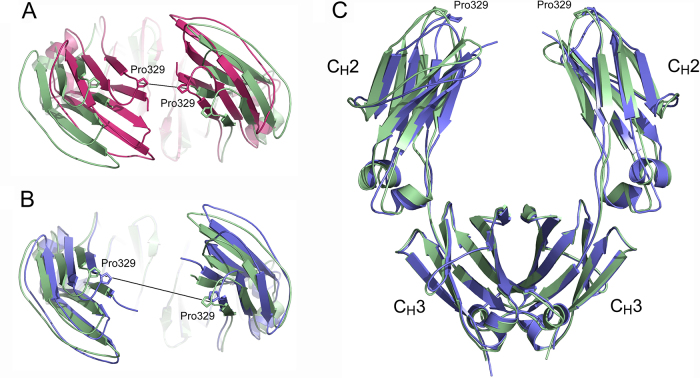
C_H_2 domain orientation. (A) View down the IgG-Fc pseudo two-fold axis with the C_H_2 domains in the foreground. The deglycosylated murine IgG1-Fc structure (PDB accession number 3HKF; Feige et al., 2009) (pink), and the Pro329 Cα atom interatomic distance (11.6 Å) indicate a closed conformation. The C_H_2 domains in the degly-Fc structure (green) adopt a more open conformation. The two Fc structures were superposed on the C_H_3 domains. (B) The deglycosylated human IgG1-Fc structure (PDB accession number 3DNK, Braden, DOI:10.2210/pdb3dnk/pdb) (blue), and the Pro329-Pro329 Cα atom interatomic distance (27.6 Å) reveals a more open conformation, similar to that for the degly-Fc structure (green). (C) Front view of the degly-Fc structure (green) and deglycosylated human IgG1-Fc structure (blue). (For interpretation of the references to colour in this figure legend, the reader is referred to the web version of this article.)

Deglycosylation of human IgG1-Fc from a decasaccharide (G2F)_2_* moiety, to a tetrasaccharide (MN2F)_2_* moiety, causes the C_H_2 domains to approach one another, and the distance between Pro329 Cα atoms, located at the top of the C_H_2 domains (Fig. 2), decreases from 33.7 Å to 21.9 Å (Krapp et al., 2003). The crystal structure of aglycosylated human IgG1-Fc (produced in *E. coli*) revealed an even more closed C_H_2-C_H_2 conformation, with Pro329-Pro329 Cα distances of 18.9 Å and 19.6 Å for the two Fc molecules of the asymmetric unit (Borrok et al., 2012), while that of deglycosylated murine IgG1-Fc was most closed, with a distance of 11.6 Å (Feige et al., 2009) (Fig. 2A). The top of one C_H_2 domain was partially disordered in the crystal structure of deglycosylated human IgG1-Fc, enzymatically treated to remove all but the first covalently attached N-acetylglucosamine moiety, but the C_H_2 domains also adopted a closed conformation; such closed conformations are incompatible with Fcγ receptor binding (Baruah et al., 2012).

In contrast, a crystal structure of fully deglycosylated human IgG1-Fc revealed an open C_H_2 domain conformation, with a Pro329-Pro329 Cα distance of 27.6 Å, stabilised by crystal packing contacts with neighbouring Fc molecules (PDB accession number 3DNK, Braden, DOI:10.2210/pdb3dnk/pdb) (Fig. 2B and C). The deglycosylated IgG4-Fc structure that we report here also adopts an open C_H_2 domain conformation, dictated by the interlocked nature of the Fc molecules in the asymmetric unit, with a Pro329-Pro329 Cα distance of 29.1 Å. This open conformation is similar to that found in the deglycosylated human IgG1-Fc structure (Fig. 2B and C), and consistent with Small Angle X-ray Scattering (SAXS) studies, where in solution, unaffected by crystal packing, the Fc molecule adopts an open conformation in the absence of oligosaccharide (Borrok et al., 2012).

### 3.4 C_H_2 domain DE loop

**Fig. 3:**
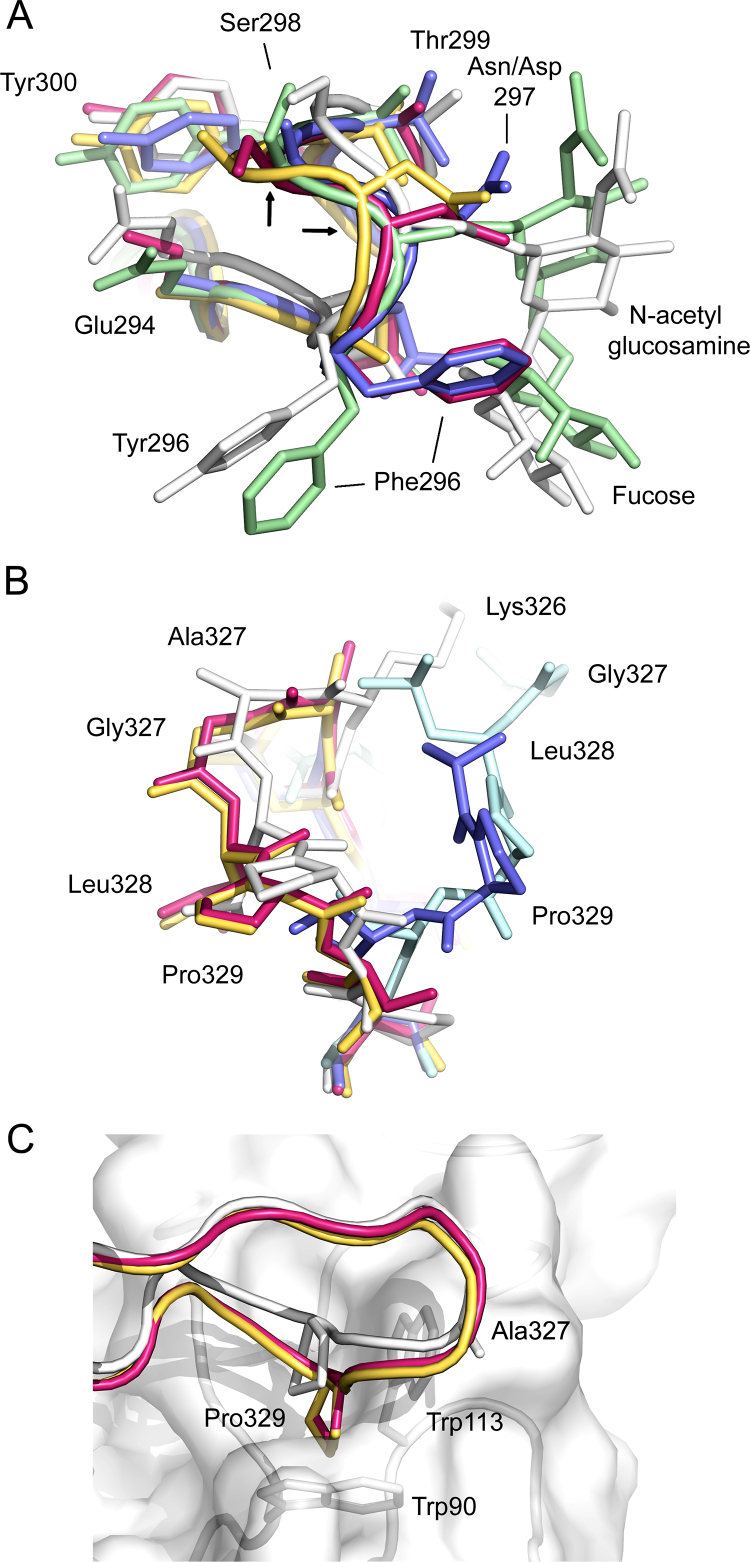
C_H_2 domain DE and FG loops. (A) The DE loop is shown for glycosylated IgG1-Fc (white) (PDB accession number 3AVE; Matsumiya et al., 2011) and glycosylated IgG4-Fc (green) (PDB accession number 4C54; Davies et al., 2014). For both glycosylated structures, the fucose residue, and the first N-acetyl glucosamine residue to which it is attached, is shown. Chains A (yellow), B (pink) and C (blue) from the degly-Fc structure are shown. The positions of the Cα atoms for residues 297 and 298 are indicated with arrows. The conformation adopted by Phe296 in molecules B and C from the degly-Fc structure clashes with the fucose residue, in contrast to the conformation adopted by Phe296 from glycosylated IgG4-Fc and Tyr296 from glycosylated IgG1-Fc, which does not. (B) The FG loop is shown for glycoslated IgG4-Fc, glycosylated IgG1-Fc and chains A-C from the degly-Fc structure. The FG loop for chains A and B from the degly-Fc structure (pink, yellow) adopt a similar conformation to the conserved loop conformation found in IgG1-Fc (white). The FG loop from molecule C (dark blue) is partially disordered, but the ordered residues adopt a similar conformation to that found in glycosylated IgG4-Fc (light blue). (C) The “proline sandwich” interaction between IgG-Fc and FcγRIII. The C_H_2 FG loop from IgG1-Fc (white) interacts with the Fcγ receptor (white), and the proline sandwich comprises Pro329 (IgG1-Fc) flanked by Trp90 and Trp113 (FcγRIII) (PDB accession number 3AY4; Mizushima et al., 2011). The C_H_2 FG loop is shown for chains A (yellow) and B (pink) from the degly-Fc structure. While the loop adopts a similar overall conformation to that in IgG1-Fc, the position of Pro329 is shifted in a manner that would clash with the receptor. (For interpretation of the references to colour in this figure legend, the reader is referred to the web version of this article.)

The C_H_2 domain DE loop, which includes Asn297 to which the oligosaccharide is attached, displays a conserved conformation in glycosylated IgG-Fc structures. It is altered, however, in enzymatically deglycosylated (Feige et al., 2009, Braden, DOI:10.2210/pdb3dnk/pdb) or aglycosylated (Borrok et al., 2012) structures. In the degly-Fc structure, reported here, the conformation of the DE loop is similarly altered (Fig. 3A). Asn297 is deamidated to Asp through the action of PNGase F, and in chains A, C and D, the Asp297 side chain adopts different rotamer positions compared with Asn297 in glycosylated IgG-Fc. Variation is also observed in the Asp297 and Ser298 Cα atom positions between the four chains of the degly-Fc asymmetric unit (up to a maximum of 1.8 Å), which both differ from their positions in glycosylated IgG-Fc (indicated by arrows in Fig. 3A).

In IgG-Fc crystal structures, Tyr296 (IgG1) or Phe296 (IgG2 and IgG4) from the DE loop adopt a range of positions, pointing towards or away from the oligosaccharide, independent of the presence or absence of a fucose residue linked to the first N-acetylglucosamine. However, in degly-Fc, Phe296 faces towards the inner face of the C_H_2 domain in a manner that would not be possible in fucosylated IgG4-Fc due to steric clashes. Adoption of this conformer facilitates the formation of van der Waals contacts between the two interlocked Fc molecules of the degly-Fc asymmetric unit (Fig. 3A).

### 3.5 C_H_3-C_H_3 interface

IgG4 undergoes a process known as Fab-arm exchange, by which the heavy chains separate to form two half-molecules; these half-molecules can then recombine with other half molecules, to form bi-specific antibodies. Together with the hinge, which forms intra- rather than inter-heavy chain disulfide bonds, Arg409 from the C_H_3 domain plays a key role in controlling Fab-arm exchange (FAE) in IgG4 (Aalberse and Schuurman, 2002, van der Neut Kolfschoten et al., 2007, Aalberse et al., 2009, Labrijn et al., 2009, Labrijn et al., 2011, Rispens et al., 2011, Rose et al., 2011). Arg409 was recently reported to adopt two different conformations at the C_H_3-C_H_3 interface; one conformer weakens the C_H_3-C_H_3 interface and disrupts a conserved network of water molecules, while the other does not (Davies et al., 2013, Davies et al., 2014). In the degly-Fc structure, the conformer which weakens the C_H_3-C_H_3 interface was only observed in chain D, while chains A, B and C contain the second, non-disruptive conformer. The co-existence of these two different conformers within a single IgG4-Fc dimer has been observed in other IgG4-Fc crystal structures (Davies et al., 2014) and provides further evidence for the flexibility of this residue.

### 3.6 C_H_2 domain FG loop

In IgG, the C_H_2 domain FG loop (residues 325–331) is important for both Fcγ receptor and C1q binding (Canfield and Morrison, 1991, Tao et al., 1993, Idusogie et al., 2000). In IgG1, the FG loop conformation is conserved, but in IgG4 it adopts a different, unique conformation, consistent with the anti-inflammatory properties of this subclass (Davies et al., 2014). In the glycosylated IgG4-Fc structure, the FG loop was unaffected by crystal packing and folded away from the C_H_2 domain; the different conformation was attributed to two sequence differences between IgG1 and IgG4, namely Ala327Gly and Pro331Ser. In contrast, the C_H_2 FG loop in chains A, B and D of the degly-Fc structure adopts a conformation which is similar to that found in IgG1, and folds back on to the C_H_2 domain. On the other hand, in chain C of the degly-Fc structure, although residues Lys326, Gly327 and the side chain of Leu328 are disordered, the positions of residues Pro329 and Ser330 are more akin to those of the unique IgG4 FG loop conformation.

In chains A, B and D the IgG1-like FG loop is stabilised by van der Waals crystal packing contacts, with C_H_3 domain residues from chains D, C and A, respectively (Fig. 1, Fig. 3). The part of the FG loop from chain C that is ordered is also stabilised by van der Waals interactions, but these are different from those for chains A, B and D. The two C_H_2 FG loop conformations observed in the degly-Fc structure demonstrate mobility of this loop in IgG4.

While the overall, conserved, conformation of the degly-Fc C_H_2 FG loop in chains A, B and D is similar to that for IgG1-Fc, the backbone atoms for residues 326–330 are shifted. In particular, compared with receptor-bound IgG1-Fc, the Cα position for Pro329 differs by ∼1.9 Å, and that for residue 327 (Gly in IgG1 and Ala in IgG1) differs by ∼1.6 Å, which would have the effect of moving the C_H_2 FG loop closer towards the Fcγ receptor (Fig. 3C). This relatively small shift in loop position is sufficient to cause interatomic clashes, and would disrupt the “proline sandwich” interaction, formed between Pro329 from the FG loop and two Trp residues from the receptor (Sondermann et al., 2000, Radaev et al., 2001, Ferrara et al., 2011, Mizushima et al., 2011, Ramsland et al., 2011).

A conserved, but shifted, C_H_2 FG loop is not unique to the degly-Fc structure as a similar perturbation is observed for IgG2, in which residue 327 is also Gly (Teplyakov et al., 2013). Much smaller perturbations of the Pro329 position are found in IgG1-Fc (e.g. PDB accession number 3DO3, Braden, DOI:10.2210/pdb3do3/pdb), suggesting a role for Ala327 in positioning Pro329 for the IgG1/Fcγ interaction.

### 3.7 IgG4-Fc can assemble into an Fc-Fc mediated hexamer

**Fig. 4:**
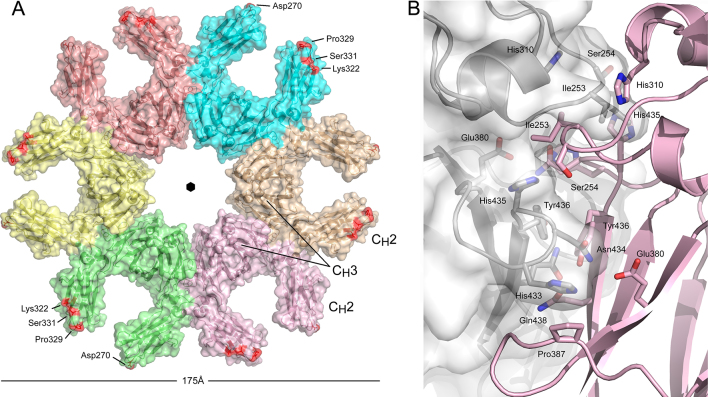
Crystallographic symmetry generates a hexameric assembly of Fc molecules. (A) Overall structure of the degly-Fc hexamer, 175 Å in diameter. Individual Fc molecules of the hexamer are coloured separately. Asp270, Lys322, Pro329 and Ser331 (Pro331 in IgG1) are coloured red, according to a model of the IgG-Fc/C1q interaction (Schneider and Zacharias, 2012). The C_H_2 domain FG loop adopts the conserved, IgG1-like conformation, compatible with C1q binding. (B) The Fc-Fc hexamer interface between chains A and B from adjacent Fc molecules, comprising residues from both C_H_2 and C_H_3 domains. The chain A Ile253 Cδ atom was not built in the degly-Fc structure, and in this figure, the same side chain rotamer observed in chain B was modelled. Chain A carbon atoms are coloured white, and chain B carbon atoms pink. (For interpretation of the references to colour in this figure legend, the reader is referred to the web version of this article.)

The C1q component of complement has a hexameric arrangement, and activates the classical complement pathway through binding to IgM and IgG immune complexes (Burton, 1990, Ghai et al., 2007, Gaboriaud et al., 2012). An IgM hexamer (or pentamer with J-chain) can activate complement (Randall et al., 1990). Recently, the involvement of IgG Fc-Fc mediated hexameric ring structures in the IgG1–Fc/C1 interaction was reported (Diebolder et al., 2014). Similar hexameric IgG Fc-Fc arrangements are found in two crystal structures of intact IgG antibodies (PDB accession number 1HZH, Saphire et al., 2001; PDB accession number 4NHH; Wu et al., 2013). The degly-Fc crystals belong to a hitherto unreported space group for IgG-Fc (*P*622), although the same space group was reported for intact IgG4 crystals (Kuznetsov et al., 2000), and the six-fold crystallographic axis generates a hexameric, planar assembly of IgG4-Fc molecules, in which chain A from one Fc molecule interacts with chain B from another (Fig. 4A).

The overall diameter of the IgG1 and IgG4 Fc hexameric rings, ∼175 Å, is similar to the diameter of the Cμ3-Cμ4 hexamer model for IgM (180 Å) (Müller et al., 2013). The IgG Fc-Fc interface involves C_H_3-C_H_3, C_H_3-C_H_2, and C_H_2-C_H_2 domain contacts (Fig. 4B), perpendicular to the six-fold rotation axis and related by a two-fold rotation, burying a surface area of approximately 2150 Å^2^. A region located on the side of the Fc molecule, at the C_H_2-C_H_3 domain interface, is involved in *Staphylococcal* protein A, *Streptococcal* protein G, FcRn, HSV-1 (herpes simplex virus 1) Fc receptor, rheumatoid factor and TRIM21 (tripartite motif 21) binding (Deisenhofer, 1981, Sauer-Eriksson et al., 1995, Corper et al., 1997, DeLano et al., 2000, Martin et al., 2001, Sprague et al., 2006, James et al., 2007). The same region facilitates other Fc–Fc interactions (Girardi et al., 2009, Kolenko et al., 2009, Davies et al., 2014), and the hexamer interface is yet another to utilise this consensus site, indirectly exploited by the C1q interaction.

The degly-Fc structure and a structure of an intact IgG antibody, both solved at 2.7 Å resolution (PDB accession number 1HZH, Saphire et al., 2001), now provide the highest resolution views of the IgG Fc-Fc hexamer interaction to date.

Residues involved in the IgG1 and IgG4 hexamer interfaces are identical. C_H_3-C_H_3 domain contact (shown in Fig. 4B) includes hydrophobic interactions between His433^A^ (chain A) and Pro387^B^ (chain B), van der Waals interactions between Tyr436^A^ and Tyr436^B^, and hydrogen bonds between Asn434^A^, Glu380^B^ and Tyr436^B^, and between Gln438^A^ and Gln438^B^ (main chain). Ile253^A^ from the C_H_2 domain protrudes into a pocket created by Leu251^B^, Ile253^B^, His310^B^, Gln311^B^, Leu314^B^ (C_H_2 domain) and His435^B^ (C_H_3 domain). Additionally, His435^A^ from the C_H_3 domain forms van der Waals interactions with Ile253^B^ and Ser254^B^ from the C_H_2 domain. Met252 from the C_H_2 domain adopts different side chain positions, but forms van der Waals interactions with nearby residues, including Met428, Asn434 and His435.

### 3.8 The C_H_2 domain AB loop in the IgG hexamer

In IgG, the C_H_2 domains adopt a range of orientations in relation to the C_H_3 domains (Teplyakov et al., 2013, Frank et al., 2014). In the degly-Fc structure, the C_H_2 domains for all chains (A–D) adopt an open conformation. Superposition of a number of IgG-Fc structures available from the Protein Data Bank onto the degly-Fc hexamer (formed by chains A and B) using C_H_3 domain Cα atoms revealed clashes between residues from the C_H_2 domain AB loop. Even superposition of chains C and D from the degly-Fc structure revealed clashes, implying that a structural change within the C_H_2 domain is required to form the hexamer.

**Fig. 5:**
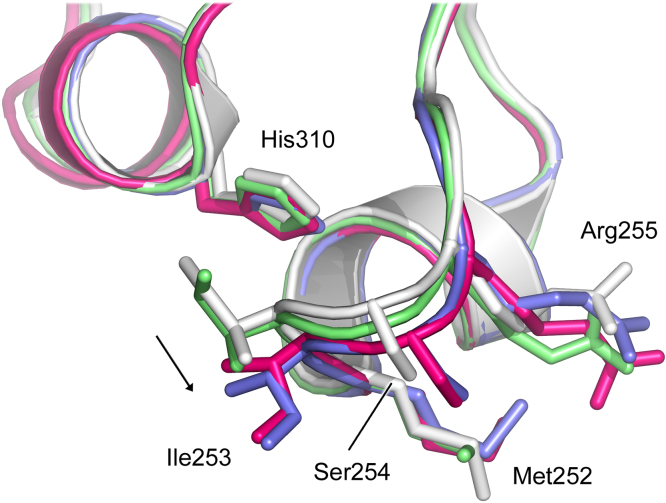
C_H_2 domain AB loop movement in hexamer formation. Ile253 and Ser254 from the C_H_2 AB loop move to accommodate formation of the IgG hexamer. The arrow close to Ile253 indicates the direction of movement. The AB loop is shown for IgG1-Fc (white) (PDB accession number 3AVE; Matsumiya et al., 2011), glycosylated IgG4-Fc (green) (PDB accession number 4C54; Davies et al., 2014), chain B of the degly-Fc structure (pink) and intact IgG1 (blue) (PDB accession number 1HZH, Saphire et al., 2001). (For interpretation of the references to colour in this figure legend, the reader is referred to the web version of this article.)

Further structural comparison, involving superposition of C_H_2 domains onto the degly-Fc hexamer, revealed close contacts mediated by Ile253 from the C_H_2 AB loop in the vast majority of structures analysed, including close contacts between Ile253^A^ and Ile253^B^ and Ile253^A^ and His310^B^, where A and B refer to interfacing chains (Fig. 4B). Thus, to form the hexamer interface, a structural change occurs in the C_H_2 AB loop, in which residues Ile253 and Ser254 move away from the C_H_2 domain. A similar loop movement is required to form both IgG1 and IgG4 hexamers, for example the Ile253 Cα atom moves 1.37 Å in IgG1 and 1.14 Å in IgG4, while the Ser254 Cα atom moves 1.53 Å (IgG1) and 1.21 Å (IgG4) (Fig. 5).

### 3.9 Implications for C1q binding

In human IgG1, residues Asp270, Lys322, Pro329 and Pro331 are important for C1q binding (Tao et al., 1993, Idusogie et al., 2000) (indicated in red on the degly-Fc hexamer in Fig. 4A; residue 331 is Ser in IgG4). In IgG4, the C_H_2 domain BC and FG loops can adopt a different conformation, disrupting the binding site (Davies et al., 2014). Formation of an IgG4 hexamer suggests that the inability of IgG4 to activate complement is the result of local structural differences in the C_H_2 domain, although the degly-Fc structure presented here does reveal a dynamic FG loop with the ability to adopt the conserved, IgG1-like conformation.

The effect of a recently described Glu345Arg mutation in enhancing complement activation (Diebolder et al., 2014) can now be rationalised through the formation of a hydrogen bond between Arg345 and the Gly385 main chain or Gln386 side chain, presumably creating a more stable hexamer. It is intriguing that this mutation increases complement activation in all IgG subclasses, suggesting that interactions at the hexamer interface are able to compensate for structural differences in the C_H_2 domain.

While residues at the IgG1 and IgG4 hexamer interfaces are identical, the interactions formed are not. For example, in both IgG1 and IgG4, variation in the Met252 side chain position is observed. Furthermore, while similar, the IgG1 and IgG4 hexamers are not identical when superposed. In a number of IgG3 allotypes, Arg435 and Phe436 replace His and Tyr, found in IgG1 and IgG4, respectively. Since these residues are both at the interface, allotypic sequence differences and variation in side chain position could affect the orientation of Fc molecules within the hexamer, which in turn might alter the disposition of the C1q binding sites, and the molecular surface presented to C1.

## 4 Conclusions

In summary, we solved the structure of enzymatically deglycosylated, serum-derived human IgG4-Fc at 2.7 Å resolution. The structure comprises a novel, interlocked arrangement of two Fc molecules and reveals structural alteration of the C_H_2 DE loop in the absence of carbohydrate. Furthermore, the C_H_2 FG loop adopts two different conformations, one unique to IgG4, and one more akin to the conserved conformation found in IgG1. Finally, crystal packing reveals a hexameric arrangement of Fc molecules, similar to the IgG1 hexamer involved in C1 binding. The deglycosylated IgG4-Fc structure reported here thus extends our understanding of this structurally less well characterised IgG subclass.
